# The effect of corticosteroids in developing active pulmonary tuberculosis among patients with COVID-19

**DOI:** 10.1371/journal.pone.0309392

**Published:** 2024-10-16

**Authors:** Thanas Praphakornmano, Pattama Torvorapanit, Noppachai Siranart, Pirapon June Ohata, Gompol Suwanpimolkul

**Affiliations:** 1 Department of Medicine, Faculty of Medicine, King Chulalongkorn Memorial Hospital, Thai Red Cross Society, Chulalongkorn University, Bangkok, Thailand; 2 Division of Infectious Diseases, Faculty of Medicine, King Chulalongkorn Memorial Hospital, Thai Red Cross Society, Chulalongkorn University, Bangkok, Thailand; 3 Center of Excellence in Tuberculosis, Faculty of Medicine, Chulalongkorn University, Bangkok, Thailand; 4 Emerging Infectious Diseases Clinical Center, Thai Red Cross Society, Bangkok, Thailand; 5 HIV-NAT, Thai Red Cross–AIDS Research Centre, Bangkok, Thailand; Children’s National Hospital, George Washington University, UNITED STATES OF AMERICA

## Abstract

Corticosteroids can reduce the mortality rate among patients with severe COVID-19 pneumonia. However, opportunistic infections such as Mycobacterium tuberculosis are of concern, especially among those on high doses of corticosteroids. It is unknown whether the risk of developing subsequent TB infection is high or not among COVID-19 patients on high doses of corticosteroids. Hence, this study was conducted to address this gap of knowledge. We conducted a retrospective, cross-sectional study at the King Chulalongkorn Memorial Hospital from October 12, 2022 to June 30, 2023. Two hundred forty-three participants with documented COVID-19 diagnosis on high dose corticosteroids were enrolled into the study. Baseline characteristics and risk factors of developing TB were collected. The prevalence of TB was significantly different among participants with chronic kidney disease (CKD) stages 2–4 and chronic lung diseases. The incidence of TB post 1-year diagnosis of COVID-19 was 4 out of 243 patients (1.6%) or 1,646 cases per 100,000 person-year. The mortality rate among subsequent TB group was significantly much higher than the non-TB group (50% vs 0.4%; p-value = 0.001). COVID-19 participants on high doses of corticosteroids also were co-infected with other infections such as bacteria (37.1%), fungi (5.3%), and *Pneumocystis jirovecii* (PJP) (1.2%). We found that the incidence of TB in participants with COVID-19 on high doses of corticosteroids was 11 times higher than the general population. Therefore, we recommend screening for latent TB among these patients to prevent/early diagnose TB disease.

## Introduction

According to the global tuberculosis (TB) report 2021 of the World Health Organization (WHO), TB was the second leading cause of deadly transmissible respiratory disease followed by COVID-19. TB was also the 13th most prevalent cause of death worldwide. In 2020, there were 1.3 million people dying from TB. As a result of this, WHO regularly sets up a goal every 5 years in reducing the incidence of TB [1].

COVID-19 is an emerging infectious disease which was first discovered on December 31, 2019, in Wuhan city, Hubei province of China. Shortly after its discovery, it had already spread rapidly to almost all of the countries around the world within a short amount of time and caused detrimental effects among those infected with the disease. Statistically, COVID-19 pneumonia is the most common cause of death at present. There were around 770 million cases and within this number, 7 million people have died from such infection (Collected information on December 6, 2023) [2]. Such high incidence of death could be a result of cytokine storm syndromes leading to severe COVID-19 disease [3]. Due to such terrifying fact, treatment with anti-inflammatory medication was studied. A case series reported good clinical outcomes in COVID-19 patients with severe pneumonia and ARDS when short course of corticosteroids was administered early [4, 5].

From one case series, the finding [6] at the King Chulalongkorn Memorial Hospital, one of the tertiary hospitals in Thailand, reported several cases of COVID-19 and Mycobacterium tuberculosis; TB infection occurred either simultaneously or subsequently within 6 months period after COVID-19 infection. In another cohort [7], 49 patients from 8 countries had TB after completing COVID-19 treatment.

In other aspects of TB risk factors, steroids also played an important role in its immunosuppressive effects on both the innate and acquired immunity [8]. Thus, many studies investigated the role of steroids and its affect in acquiring TB infection. One study reported that patients on steroids had higher rates of bacterial and fungal infections [9]. Another study discovered that both concurrent use of steroids and past use of steroids within 3–12 months were significantly correlated with increased incidence of TB infection [10]. In contrast, in another study, it was reported that a lower dose of steroids, which was equivalent to the cumulative dose of prednisolone 700 mg, had no effect on the immune system and did not increase the risk of acquiring infection(s) [11]. In addition, the consensus guideline of American Thoracic Society (ATS) and Center for Disease Control and Prevention (CDC) suggest that people taking only prednisone 15 mg/day continuously for 2–4 weeks were at an increased risk for acquiring TB infection [12].

Therefore, we assessed the incidence of TB among people who were infected with severe COVID-19 and received high dose corticosteroids. The primary objective assessed the TB incidence within 12 months among COVID-19 patients on corticosteroids. The secondary objectives assessed the incidence of other infections such as bacteria, fungi, *Pneumocystis jirovecii* pneumonia (PJP) and non-tuberculous mycobacteria (NTM) as well as incident TB infection after completing a course of COVID-19 treatment.

## Material and methods

We conducted a retrospective, cross-sectional study at the King Chulalongkorn Memorial Hospital from October 12, 2022 to June 30, 2023. The identifications of the individual participants were not available during or after data collection. The authors reviewed the electronic medical records (EMRs) and screened for patients that had ICD-10 codes such as J00, J028 and/or J128 which were interpreted as any respiratory tract infection(s) combined with another code, U071 or SARS-CoV-2 infection. COVID-19 infection was detected by collecting specimens from nasopharyngeal swab, throat swab or sputum and RT-PCR method was performed to confirm the diagnosis.

The participants were then screened for dexamethasone use through ICD-10 coding with 3DEXA5 or 3LODEXA5 since dexamethasone was used to treat COVID-19 pneumonia according to the concurrent Thai guideline. The investigator calculated the dose of dexamethasone by using the conversion factor of dexamethasone 0.75 mg to prednisolone 5 mg [13].

The inclusion criteria for this study were as follows: 1) Age 18 years or older, 2) Diagnosis and positive test results for COVID-19 using RT-PCR for SARS-CoV-2, and 3) Receiving dexamethasone with an equivalent dosage to cumulative amount of prednisolone 700 mg or prednisolone 15 mg/day used continuously for 14 days or more during COVID-19 pneumonia treatment course. The investigator defined such dosage of steroids as “high dose steroids” in this study.

The exclusion criteria were: 1) patients with concurrently active TB before COVID-19 diagnosis, 2) patients who died from causes other than TB infection within 12 months after enrollment, and 3) patients who did not receive high doses of steroids as defined in the study.

Regarding statistical analysis, IBM SPSS Statistics version 28 program was used. Chi-square test or Fisher exact test was used to compare the differences between TB and non-TB groups.

In terms of the ethics statement, the patients’ data were encrypted and de-identified as per the Thai Personal Data Protection Act 2019, Thailand (PDPA). The study was approved by the Institutional Review Board (IRB No. 0630/65), Faculty of Medicine, Chulalongkorn University on October 12, 2022. Data was extracted from the database on October 12, 2022 until February 28, 2023. Data was analyzed from February 28, 2023 until June 30, 2023. Informed consent was waived because this is a retrospective study and we did not see the patients. Data was retrospectively analyzed. ChatGPT was not used in this study.

### Theory/Calculation

We have consulted with the statistician. Since this is a retrospective study, thus there is no need for sample size calculation.

## Results

**Fig 1:**
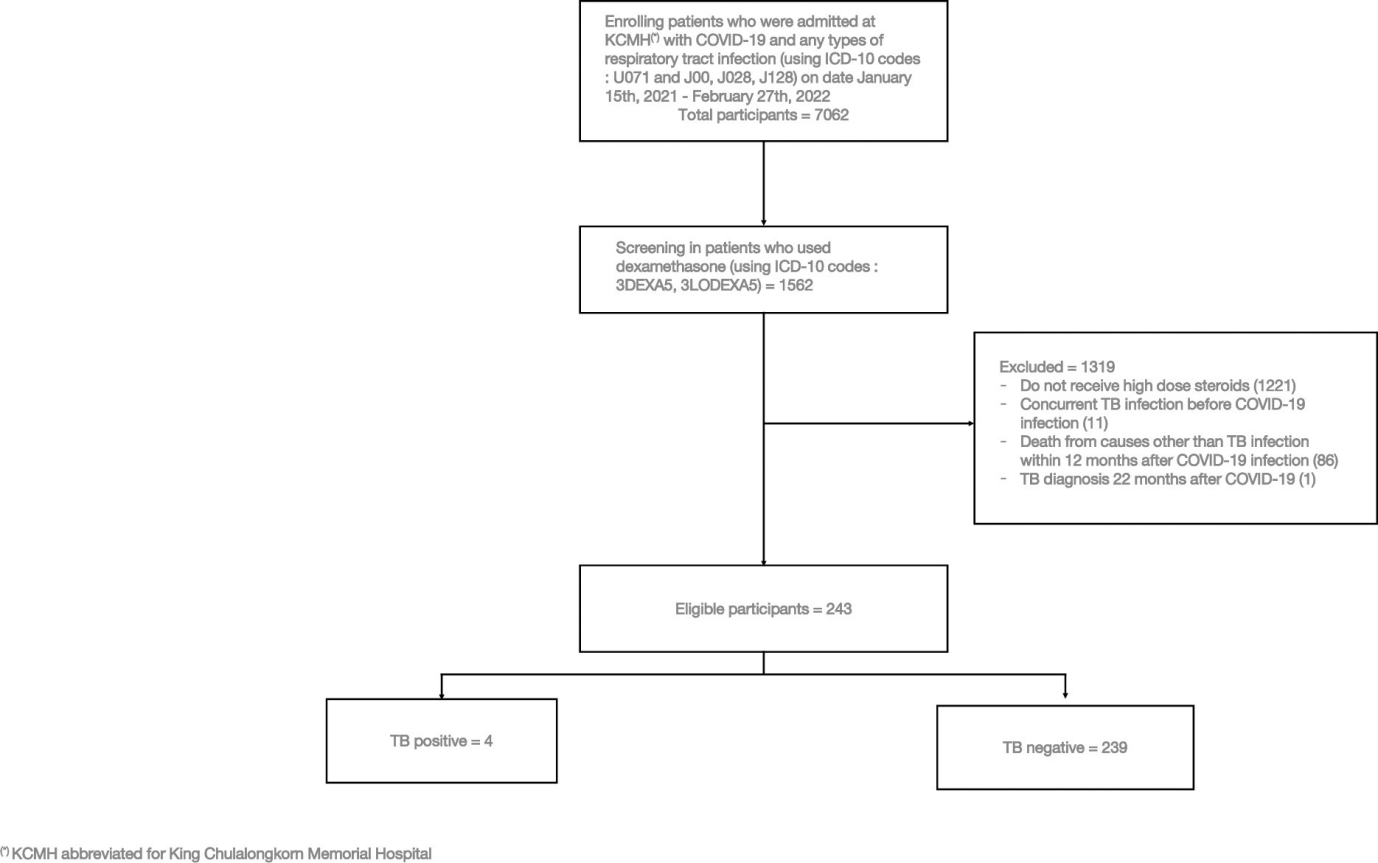
Flow chart.

The electronic medical records of 7062 patients from in-patient departments of the King Chulalongkorn Memorial Hospital with ICD-10 codes of COVID-19 (U071) and any types of respiratory tract infection(s) (J00, J028, and/or J128) since January 15, 2021, to February 27, 2022, were reviewed and screened for eligibility to enter the study. After the initial screening, those patients were then screened for dexamethasone use (3DEXA5, and 3LODEXA5) which gave us 1562 patients to analyze. 1317 patients were excluded from the analysis as follows: 1221 patients did not receive high dose steroids, 9 patients had concurrent TB before COVID-19 diagnosis, 86 patients were dead from other causes than TB infection, and 1 patient was diagnosed with TB out of the study time-frame (TB diagnosis more than 12 months after COVID-19 diagnosis) (Fig 1).

In regards to the outcome findings, we defined subsequent TB group as patients who were diagnosed with TB within 12 months after COVID-19 diagnosis and non-TB group as patients who were not diagnosed with TB within such time frame. As for the baseline characteristics, we collected the following variables: sex, age, body mass index (BMI), underlying diseases including diabetes mellitus, chronic kidney disease (CKD), chronic lung diseases (specifically chronic obstructive pulmonary disease (COPD)), HIV infection, autoimmune diseases, organ transplant status, immunosuppressive drug use and history of previous TB infection as shown in Tables 1 and 2.

**Table 1:** Characteristics of the participants.

	Total (N = 243)	non subsequent TB (N = 239)	Subsequent TB (n = 4)	P-value
Sex: male, n (%)	128 (52.7)	124 (51.9)	4 (100)	0.22
Age (years), median (IQR)	66 (57–75)	66 (56–74)	77.5 (72.5–82.5)	0.04
Age group, n (%)				0.050
•< 75 years	182 (75.7)	181 (75.7)	1 (25)	
•≥ 75 years	61 (24.3)	58 (24.3)	3 (75)	
BMI (kg/m^2^), median (IQR)	27.1 (23.8–31.4)	27.1 (23.8–31.4)	24.3 (23.1–27.7)	0.09
BMI group, n (%)				0.11
•< 25	82 (33.7)	79 (33.1)	3 (75)	
•≥ 25	161 (66.3)	160 (67)	1 (25)	
Diabetes mellitus, n (%)	107 (44)	104 (43.5)	3 (75)	0.32
CKD stage, n (%)				0.002
•0–1	223 (91.8)	222 (92.9)	1 (25)	
•2–4	20 (8.2)	17 (7.1)	3 (75)	
Chronic lung disease, n (%)				0.001
•No	229 (94.2)	228 (95.4)	1 (25)	
•Yes	14 (5.8)	11 (4.6)	3 (75)	
COPD, n (%)				0.007
•No	234 (96.3)	232 (97.1)	2 (50)	
Yes	9 (3.7)	7 (2.9)	2 (50)	
Autoimmune disease, n (%)	2 (0.8)	2 (0.8)	0 (0)	0.85
Organ transplant status, n (%)	2 (0.8)	2 (0.8)	0 (0)	0.85
Previous TB, n (%)	4 (1.7)	4 (1.7)	0 (0)	0.79
HIV, n (%)	4 (1.7)	4 (1.7)	0 (0)	0.79
Smoking, n (%)	30 (12.4)	26 (10.9)	4 (100)	<0.001
Alcohol, n (%)	11 (4.5)	11 (4.6)	0 (0)	0.66
Immune suppressive drug, n (%)				0.28
•No med	170 (70)	168 (70.3)	2 (50)	
•Tocilizumab	48 (19.8)	47 (19.7)	1 (25)	
•Tofacitinib	17 (7)	16 (6.7)	1 (25)	
•Other drugs	8 (3.3)	8 (3.4)	0 (0)	
Death	3 (1.2)	1 (0.4)	2 (50)	0.001

Chi-square test or fisher exact test was used to compare between the TB group and non-TB group. Continuous data among the groups were compared using Wilcoxon rank sum test.

**Table 2:** Risk factors associated with subsequent tuberculosis.

	Univariate	
	OR (95%CI)	P-value
Age		
•< 75	1	Ref
•≥ 75	9.36 (0.96–91.75)	0.06
BMI group		
•< 25	6.07 (0.62–59.4)	0.12
•≥ 25	1	Ref
Diabetes mellitus: Yes vs No	3.89 (0.40–37.98)	0.24
CKD stage		
•0–1	1	Ref
•2–4	39.2 (3.86–397.2)	0.002
Chronic lung disease: Yes vs No	62.2 (6.0–647)	0.001
COPD: Yes vs No	33.1 (4.1–270)	0.001
Immune suppressive drug		
•No med	1	Ref
•Tocilizumab	1.78 (0.16–20.14)	0.64
•Tofacitinib	5.25 (0.45–61.1)	0.18

OR: Odd ratio, aOR: Adjusted Odd ratio, 95% CI: 95% confidence interval

From a total number of eligible 243 participants, there were 128 (52.7%) males at birth and 115 (47.3%) females at birth; 124 (51.9) were males at birth in the non-TB group and 115 (48.1%) females at birth in the non-TB group. There were 4 (100%) subsequent TB cases in the study, all of them were males at birth. As for the median age of the non-TB group, it was 66 years, whereas subsequent TB group was 77.5 years and the difference was statistically significant (p-value = 0.04). This was in accordance with the subgroup finding that subsequent TB infection was prevalent among patients aged ≥ 75 years (75% vs 25%, p-value = 0.05).

From a total number of 243 participants, the median for BMI was 27.1 kg/m^2^ (IQR 23.8–31.4 kg/m^2^) and 33.7% of the participants had BMI < 25 kg/m^2^. Most of the TB participants had BMI < 25 kg/m^2^ (75%), however this was not statistically significant (p-value = 0.11). Aside from that, 44% of the participants had diabetes and 75% of the participants in the subsequent TB group had diabetes but this was not statistically significant when compared to the non-diabetic participants (p-value 0.32). Even though there were few participants with CKD stages 2–4 (according to KDIGO 2023 classification), we found that these participants were more likely to develop TB compared to non-CKD participants (75% vs 25%, p-value = 0.002). Also, the participants with chronic lung diseases (asthma, COPD, and bronchiectasis) developed TB more than non-chronic lung disease participants (75% vs 25%, p-value = 0.001).

On the other hand, there was no difference in the non-TB group and subsequent TB group for the presence of COPD as a subset group of chronic lung diseases (9 out of 14 participants (64.3%) with chronic lung diseases). Participants with autoimmune diseases (rheumatoid arthritis, systemic lupus erythematosus (SLE), and polyarteritis nodosa), organ transplants (kidney transplant, and hematopoietic stem cell transplant), HIV infection or participants with alcohol drinking history did not develop incident TB; however, the statistical difference was not significant in the subsequent TB group and non-TB group. As of note, 30 of 243 eligible participants (12.3%) had a history of smoking and all of them had subsequent TB which indicated that smoking was a significant risk factor for developing TB (100%, p-value < 0.001).

The co-administration of immunosuppressive drugs were tocilizumab (19.8%), followed by tofacitinib (7%), and other drugs (3.3%) including baricitinib, sulfasalazine, mycophenolate mofetil, tacrolimus, hydroxychloroquine and chemotherapy, and mixed groups of drugs (0.3%).

The incidence of subsequent TB was 25% in tocilizumab group and 25% in tofacitinib group compared to 50% in the non-immunosuppressive drug group; however, there was no statistical difference among participants with and without immunosuppressive drug(s) (p-value = 0.28).

The mortality rate among subsequent TB group was significantly much higher than those in the non-TB group (50% vs 0.4%; p-value = 0.001). As for post-COVID-19 treatment, four participants were diagnosed with TB at 9 days, 2 months, 4 months, and 7 months, respectively. The incidence of subsequent TB occurred in 4 out of 243 participants (1.6%, 95% CI: 0.4–4.2) or could be inferred as 1646 cases per 100,000 person-year and this information is shown in Table 3.

**Table 3:** Details of the four subsequent TB participants.

Case Number	Sex	Age	Underlying diseases	Steroid dose	Other immunosuppressive drug use	Timing of active TB after COVID-19 infection	Imaging study	Microbiological lab diagnosis	Treatment	Complication	Outcome
**1**	Male	76	Colon cancer status remission, diabetes, hypertension, CKD stage 3B	1200 mg	Tocilizumab	9 days	Cavity at RUL with surrounding consolidation	Sputum PCR for MTBC positive, no drug resistance	IREL for 1 day (due to mild transaminitis)	P.aeruginosa sepsis, Candidemia, Pulmonary embolism	Dead within the admission
**2**	Male	86	Coronary artery disease status PCI, BPH, diabetes, hypertension, CKD stage 3B	2930 mg	Tofacitinib	2 months	GGO with consolidation at LUL	Sputum AFB/PCR for MTBC/Culture TB all positive, no drug resistance	2IRZE/4IR	HAP, Organizing pneumonia, E.Coli UTI, Pulmonary embolism	Cured
**3**	Male	79	Pulmonary hypertension, COPD GOLD E, Myelofibrosis, Prostate cancer, CKD stage 3B	840 mg	None	4 months	Massive right pleural effusion	Pleural fluid culture TB positive, no drug resistance	2IRZE, then IR for 2 months	A.baum HAP, E.Coli UTI, Cellulitis	Dead in the later admission from the cause of worsening PH and RV failure
**4**	Male	69	Diabetes, dyslipidemia	2420 mg	None	7 months	Reticulopatchy consolidation with multiple solid nodules at RUL	Sputum AFB/PCR for MTBC/geneXpert all positive, no drug resistance	2IRZE/4IR	HAP, Pulmonary embolism	Cured

Overall, co-infection accounted for 35.4% of which the most common pathogens were bacteria. 90 out of 243 patients (37.1%) had bacteria which was followed by fungi in 13 out of 243 patients (5.3%) and PJP in 3 out of 243 patients (1.2%); none of the participants were infected with nontuberculous mycobacteria (NTM). For bacterial co-infection, the top 3 most common identified organisms were *K*.*pneumoniae* (4.5%), *E*.*coli* (4.1%) and *P*.*aeruginosa* (3.7%). Only two species of fungi were detected as co-infection in this study, Aspergillus spp. and Candida spp., of which Aspergillosis occurred more often than *Candida* infection (58.3% vs 41.7%). In terms of co-infection, the most prevalent infection was pneumonia (14.8%), followed by urinary tract infection (UTI) (4.5%) and catheter-related bloodstream infection (CRBSI) (1.6%).

## Discussion

Regarding baseline characteristics, firstly, we found that there was a trend of higher incidence of TB among the elderlies aged ≥ 75 years.

In terms of BMI, unlike in the previous study [14] that suggested underweighting as a risk factor of TB, BMI difference with a cut-off at 25 kg/m^2^ in our study did not appear to have any statistical difference in developing TB.

In a previous study, diabetes was considered to be one of the risk factors for developing TB infection [14]; however, we found it to be a non-significant risk factor for developing TB. Autoimmune diseases, organ transplants, HIV infection, previous TB infection or alcohol drinking history were also not found to be risk factors for developing TB in our study. Contrary to these results, one large retrospective cohort study [15] had shown that male sex, age > 40 years, diabetes mellitus, COPD, HIV infection and any cancer were associated with subsequent TB disease. Thus, due to the very low number of positive TB cases in our study, we could not judge if the aforementioned risk factors are associated with the risk of developing subsequent TB or not. Additional study with a larger sample size is needed to address this adequately.

Smoking was found to be in accordance with the previous study [14] which indicated that it was one of the risk factors for developing subsequent TB.

Chronic kidney disease and chronic lung diseases were associated with TB infection. The results were concordant with the previous study [14] which suggested end-stage renal disease (ESRD) was a high-risk factor for developing TB.

In terms of the primary outcome, TB incidence in the general population in Thailand in 2020 [16] was 150 cases per 100,000 person-year. As for this study, the TB incidence was 4 participants out of 243 or could be inferred as 1646 cases per 100,000 person-year (11 times more than the Thai general population). This result was consistent with another similar study [15] which reported that symptomatic COVID-19, especially pneumonia, increased the risk for developing subsequent TB disease within 300 days of follow-up.

In regard to mortality among subsequent TB participants in our study, two were dead. We found out that these two were elderly male participants aged ≥ 75 years and had developed TB after COVID-19 in less than 4 months (9 days and 4 months, respectively) and one was given tocilizumab. These results were in accordance with the recent study [17] which revealed that with increasing age, having a shorter time interval to develop TB after COVID-19 diagnosis (especially within 90 days) and being immunocompromised were significant risk factors of mortality in TB and COVID-19 co-infected patients.

To the best of our knowledge, this is the first study to demonstrate that the effect of corticosteroids in patients with COVID-19 increased the risk for acquiring opportunistic infection, particularly TB, aspergillosis, and *Pneumocystis jirovecii* (PJP*)*. We also demonstrated that there was an increased incidence of active TB after the patient initiated steroid by more than 11 times compared to the general population. We were also able to ascertain the risk factors for developing active pulmonary TB. COVID-19 associated pulmonary aspergillosis (CAPA) and PJP are also important opportunistic infections that are of concern. Furthermore, additional research is needed to determine who should receive primary prophylaxis to prevent PJP or who are at risk to develop CAPA. However, there were some limitations in our study. First, when we screened the patients, we found that the number of medications allocated, and the number of drugs prescribed were not in line for some patients (data examination via the hospital system’s software). Despite this limitation, there was no inter-observer variability since we had only one investigator who screened the patients for eligibility.

Second, many potential participants may have been excluded by the criterion of not receiving high dose steroids (1221 participants were screened out). We constructed such criterion at first with the expectation that a greater number of subsequent TB infection would be found.

Lastly, because PCR test for PJP had high sensitivity (96.9%) and high specificity (94.6%) of detection [18], it was employed as standard diagnostic test in our hospital. However, the positive PCR test from BAL or sputum (the specimen was collected in such fashion in our participants) could only be presumed as probable PJP disease according to the consensus definitions from European Organization for Research and Treatment of Cancer and the Mycoses Study Group Education and Research Consortium, so the incidence found in our study should be interpreted with caution [19].

## Conclusions

From the findings, we conclude that the incidence of TB in patients diagnosed with COVID-19 who received high doses of steroids is 11 times more than the Thai general population. Moreover, the mortality rate among subsequent TB group was significantly much higher than the non-TB group. Thus, screening protocol for latent TB such as Interferon-Gamma Release Assays (IGRAs) may be beneficial in this group of patients, especially in patients with CKD and chronic lung diseases to prevent subsequent TB disease and reduce the disease severity and mortality among patients with TB and COVID-19 coinfection.

## Supporting information

S1 Checklist Human participants research checklist. (DOCX)

S1 Raw data (XLSX)

S2 Raw data (XLSX)
